# Altered Expression of Cyclin A 1 In Muscle of Patients with Facioscapulohumeral Muscle Dystrophy (FSHD-1)

**DOI:** 10.1371/journal.pone.0073573

**Published:** 2013-09-03

**Authors:** Anna Pakula, Joanna Schneider, Jürgen Janke, Ute Zacharias, Herbert Schulz, Norbert Hübner, Anja Mähler, Andreas Spuler, Simone Spuler, Pierre Carlier, Michael Boschmann

**Affiliations:** 1 Experimental and Clinical Research Center, a joint co-operation of Charité University Medicine and Max-Delbrück Center for Molecular Medicine, Franz-Volhard Center for Clinical Research, Berlin, Germany; 2 Experimental and Clinical Research Center, a joint co-operation of Charité University Medicine and Max-Delbrück Center for Molecular Medicine, Muscle Research Unit, Berlin, Germany; 3 Max-Delbrück Center for Molecular Medicine, Department of Molecular Epidemiology, Berlin, Germany; 4 Max-Delbrück Center for Molecular Medicine, Department of Experimental Genetics of Cardiovascular Diseases, Berlin, Germany; 5 Klinik für Neurochirurgie, HELIOS Klinikum Berlin-Buch, Berlin, Germany; 6 Institut de Myologie, AIM-CEA NMR Laboratory, Institute of Myology, Pitie-Salpetriere University Hospital, Paris, France; University of Canberra, Australia

## Abstract

**Objectives:**

Cyclin A1 regulates cell cycle activity and proliferation in somatic and germ-line cells. Its expression increases in G1/S phase and reaches a maximum in G2 and M phases. Altered cyclin A1 expression might contribute to clinical symptoms in facioscapulohumeral muscular dystrophy (FSHD).

**Methods:**

Muscle biopsies were taken from the *Vastus lateralis* muscle for cDNA microarray, RT-PCR, immunohistochemistry and Western blot analyses to assess RNA and protein expression of cyclin A1 in human muscle cell lines and muscle tissue. Muscle fibers diameter was calculated on cryosections to test for hypertrophy.

**Results:**

cDNA microarray data showed specifically elevated cyclin A1 levels in FSHD vs. other muscular disorders such as caveolinopathy, dysferlinopathy, four and a half LIM domains protein 1 deficiency and healthy controls. Data could be confirmed with RT-PCR and Western blot analysis showing up-regulated cyclin A1 levels also at protein level. We found also clear signs of hypertrophy within the *Vastus lateralis* muscle in FSHD-1 patients.

**Conclusions:**

In most somatic human cell lines, cyclin A1 levels are low. Overexpression of cyclin A1 in FSHD indicates cell cycle dysregulation in FSHD and might contribute to clinical symptoms of this disease.

## Introduction

Facioscapulohumeral muscular dystrophy (FSHD) is an autosomal dominant neuromuscular disorder. It is the third most common hereditary muscle disease with an estimated incidence of 1∶20,000. FSHD usually begins in adulthood and is foremost characterized by progressive and asymmetrical weakness and wasting of specific muscles of the face, shoulder girdle and upper arms, but may progress also to the lower legs [1]–[4]. There are two types of FSHD: FSHD 1 (classic one) and FSHD-2. Both are clinically identical and the only difference results from genetic background. FSHD-1 is associated with contractions of an integral number of 3.3 kb KpnI (D4Z4) macrosattelite repeats in the subtelomeric region of the long arm of chromosome 4 (4q35). D4Z4 repeats consist of 11–100 KpnI units in healthy subjects and FSHD-2 patients, but only 1–10 KpnI units in FSHD-1 patients. The most frequent haplotype is 4qA161 [1]–[5]. Recently, Lemmers et al. reported that digenic inheritance of an SMCHD1 (encoding structural maintenance of chromosome flexible hinge domain containing 1) mutation and an FSHD-permissive D4Z4 allele causes FSHD-2 [6].

FSHD is not only related to D4Z4 contractions but is also associated with up-regulation of some genes proximal to the deletion, including FSHD region gene 1 (*FRG1*) and 2 (*FRG2*), and adenine nucleotide translocase-1 (*ANT-1*). *FRG1* encodes a RNA splicing regulator and *FRG2* protein is related to RNA biogenesis. ANT-1 is a Ca^2+^-dependent protein and a component of the mitochondrial permeability transition pore (MPTP). It plays an important role in the regulation of oxidative phosphorylation [1], [3]–[5], [7]. Moreover, over-expression of ANT-1 as well as the deficiency of complex III of the mitochondrial respiratory chain suggest that FSHD is also associated with mitochondrial dysfunction [8]. Over-expression of ANT-1 leads to the opening of mitochondrial permeability transition pore and efflux of calcium ions from the mitochondria leading finally to apoptosis [1]–[5], [7]–[10].

Earlier studies revealed different aspects associated with FSHD including cell cycle dysregulation [11]. Progression of cells through the cell cycle is controlled by cyclins, a family of proteins activating cyclin-dependent kinases (CDK). One of these cyclins, cyclin A1 (CCNA1) phosphorylates both CDK1 and CDK2, resulting in two distinct kinase activities- one appearing in S phase, the other in G2 - and hence regulating transition between cell cycle phases. Several authors have shown that overexpression of *CCNA1* may cause chromatin condensation, dysregulated double strand break repair and, consequently, apoptosis. Therefore, up-regulation of *CCNA1* might lead to similar results in FSHD [12]–[16]. Moreover, cyclin A1 is normally suppressed or expressed on a low level in most somatic cells [17]. Recently, two independent research groups have identified cell cycle dysregulation in FSHD by gene expression profiling. Both FSHD-1 and FSHD-2 cells show common and distinctive dysregulation in gene expression pattern and alterations in cell cycle control. Interestingly, FSHD-1 myoblasts (when compared to healthy control cells) showed dysregulation in cell cycle activity and proliferation processes whereas FSHD-2 myotubes are mainly linked to dysregulated RNA processing. Transcriptional profiles of several genes have been also investigated in human muscle biopsies selected according to different MRI patterns. In FSHD muscles, myopathic and inflammatory changes are characterized by increased signals of T2 - short *tau* inversion recovery (T2-STIR) sequences (also in muscle not yet replaced by fat tissue). Normal healthy muscle does not present elevated T2 values. Following alterations in muscle regeneration (derived only from muscle with elevated T2 which indicates T2-STIR hyperintensity), up-regulation of *CCND1, CCND2, CCND3, CCNA1* and *CDK 4* and *CDK6* and down-regulation of *CDKN1B* were found [18]. In this article we present evidence of cyclin A1 overexpression at both RNA and protein level in FSHD-1, but not in other muscular dystrophies such as caveolinopathy 3 (CAV 3), dysferlinopathy (DYSF) and four and a half LIM domains protein 1 deficiency (FHL1).

## Subjects and Methods

### Ethics Approval

Our study was approved by the local Ethics Committee of the Charité University Medicine Berlin (EA1/166/09) and written informed consent of participants was obtained before entry into the study.

### Muscle Biopsies

Open muscle biopsies were obtained from the *Vastus lateralis* muscle of seven FSHD patients and 30 control subjects (19 healthy controls for all experiments and 11 patients with defined muscular disorders) for microarray analysis. Healthy controls had no muscle weakness and no evidence for neuromuscular disorders (displayed normal creatine kinase level and normal muscle histology). Biopsy specimens were immediately flash-frozen under cryoprotection and then used for cryosectioning, staining, calculation of fiber diameter area, total RNA isolation and quantitative real-time (RT)-PCR. Cryosections were subjected to routine staining protocols such as H&E and Gomori Trichrome.

### Cell Culture

Primary myoblast cultures were isolated from fresh muscle biopsies from seven FSHD-1 patients and from fourteen healthy control subjects (seven of them were age-matched) as previously described [19]. Cultures were enriched for myoblasts by magnetic cell sorting and the use of anti-CD56 to reach at least 90% of myoblasts (Miltenyi Biotech, Bergisch Gladbach, Germany). Differentiation of myoblasts into myotubes was initiated at approximately 90% confluence by switching to differentiation medium containing DMEM (GIBCO, Darmstadt, Germany) and 2% horse serum. All experiments were carried out using cell lines between 2 and 10 population doublings to avoid premature replicative senescence which usually appears after 10–15 population doublings [11]. Cells were collected after 3–7 days of differentiation when about 80–90% of mononuclear myoblasts had fused to form multinuclear elongated myotubes [19], [20]. All data regarding FSHD primary muscle cells are summarized in Table 1.

**Table 1:** Characterization of FSHD-1 patients.

Patientnumber	Age in years	Gender	*Eco* RI+fragment (kb)	*Eco* RI+ *Bln* I fragment (kb)	MRCS (1–5)	MB&MT culture	RNA MB&MT	Proteins (MT)	RNA(muscle biopsy)	Proteins(muscle biopsy)	Hypertrophy
1	22	M	34	31	4	+	+	+	−	+	+
2	54	M	33	30	4	+	+	+	+	+	−
3	42	M	32	29	4	+	+	+	+	+	+
4	37	M	27	24	4	+	+	+	−	−	+
5	66	F	27	24	4	+	+	−	+	−	+
6	72	F	37	34	4	+	+	−	−	−	−
7	57	F	15	12	4	+	+	−	−	−	+

Numbers of patients correspond to the number shown on the presented Western blot figures. MRCS (Medical Research Council Scale) is a system for grading muscle strength from 0 to 5. (0- Zero, 1- Trace, 2- Poor, 3- Fair, 4-Good, and 5- Normal). RNA was isolated form myoblasts (MB), myotubes (MT) and muscle tissue. Protein samples are from MT and muscle tissue. Gender of patients is indicated as “m” for male and “f” for female. (+) indicates if a parameter/sample was taken and (-) when not.

### RNA Isolation and Quantitative Real-time Reverse Transcriptase PCR (TaqMan)

Total RNA was isolated with the Qiagen RNeasy mini kit including the RNase-Free DNAse set (Qiagen, Hilden, Germany) according to the instruction of the manufacturer. RNA (1 µg) was reverse transcribed into cDNA by using the High Capacity cDNA Reverse Transcription Kit (Applied Biosystems, Darmstadt, Germany) and analyzed by real-time quantitative PCR on an ABI 7500 Fast Real-time PCR system (PE Biosystems). PCR reactions were carried out using Taqman PCR Mastermix (Applied Biosystems, Darmstadt, Germany) and TaqMan Gene Expression Assays (Hs00171105_m*, Applied Biosystems, Darmstadt, Germany). Quantitative RT-PCR was performed as follows: 2.5 µL of master mix (2x), 0.25 µL primer assay (20x) and 1 µL template cDNA (10 ng/ul) and 1.25 µL H20 were added to each well. After brief centrifugation, the PCR plate was subjected to thermocycling for 40 cycles with PCR activation at 95°C for 2 min, denaturation at 95°C for 3 seconds, and annealing/extension at 60°C for 20 s. All samples and controls were run in triplicates. Quantitative RT–PCR data were analyzed by the comparative cycle number threshold method. Results are shown as the ratio of the reference gene to target gene by using the formula: ΔCt = Ct (target genes) − Ct (*18S*). To determine the relative expression levels, we used: ΔΔCt = ΔCt (FSHD) – ΔCt (Control). The n-fold difference was determined by the expression 2^−ΔΔCt^.

### RNA Isolation and Microarray

Total RNA was isolated from myotubes derived from different cell lines for DYSF (n = 4), CAV3 (n = 4), FSHD (n = 4), FHL1 (n = 3), and healthy controls (n = 7) by using the standard TRIzol reagent (Invitrogen, Darmstadt, Germany) according to the manufacturer’s instructions. cDNAs were synthesized using the Ambion's WT expression kit (http://www.ambion.com/). Fragmentation and labeling was done by using the standard Affymetrix protocol. Fragmented cDNA was hybridized for 16 h at 45°C (Gene Chip hybridization oven 640) to the human exon array. Arrays were washed and stained with streptavidin-phycoerythrin in the Affymetrix Fluidics Station 450 and further scanned using the AFFYMETRIX GeneChip Scanner 3000 7G (http://www.affymetrix.com). The image data were analyzed with the Gene Chip command console using Affymetrix default analysis settings.

### Analysis of Expression Data

Arrays were quantile-normalized with respect to the probe GC content using the Robust Multi-array Average (RMA) algorithm (GC content adjustment, RMA background correction and mean probe set summarization). No or low expressed transcripts (max. native signal <100) were removed. Data filtering led to a set of 25,271 meta-probe sets. Differential expression was tested by using ANOVA followed by controlling the false discovery rate (FDR) according to Benjamini et al. [21]. We found 59 differentially expressed probe sets between the five sample groups (5% FDR).

The data discussed in this publication have been deposited in NCBI's Gene Expression Omnibus [22] and are accessible through GEO series accession number GSE44874.

### Immunofluorescence and Immunohistochemistry

For immunofluorescence (IF), cells were fixed 48h after seeding with 3.7% paraformaldehyde for 20 min and then permeabilized with 0.2% Triton X-100 for 15 min, followed by blocking with 1% bovine serum albumin (BSA) for 30 min. Then, the primary antibodies rabbit anti-cyclin A1 (Abcam), and mouse anti-desmin (DAKOcytomation, Hamburg, Germany) were added and incubated at room temperature for 1 h. Immunohistochemistry (IH) was performed on 6 or 10 µm frozen sections of *Vastus lateralis* muscle from eight FSHD patients and eight age-matched healthy controls. Sections were fixed in 100% acetone for 5 min and then blocked with 1% BSA for 30 min. Then, the primary antibodies rabbit anti-cyclin A1 (Abcam), and mouse anti-dystrophin (C-terminus) NCL-DYS2 (Novocastra Labolatories, Wetzlar, Germany) were added and incubated at room temperature for 1 h. For both, IF and IH, the secondary antibodies Alexa594-conjugated anti-mouse IgG or Alexa488-conjugated anti-rabbit IgG (Invitrogen, Cergy Pontoise, France) were added and incubated for another 30 min. Hoechst (Sigma-Aldrich, Hamburg, Germany) was used for 3 min to stain nuclei. Fluorescence images were captured by using a fluorescent microscope (Leica Microsystem LAS AF, AF 6000 Modular Systems).

### Western Blot

Proteins were isolated from myotubes by solubilization in ice-cold lysis buffer (50 mM TRIS, 150 mM NaCl, 0.5% Triton, 0.5% Na-Deoxycholate, 50 mM NaF, 1 mM Vanadate, pH = 7.4, containing protease inhibitors, complete EDTA free; Roche, Mannheim, Germany). Protein lysates were separated with 10% SDS-Page gel and transferred to a nitrocellulose transfer membrane (Whatman, Dassel, Germany). The primary antibodies for CCNA1 (BD) and *β*-tubulin (Abcam) were diluted in 4% milk-powder in TBS with 0.05% Tween (TBS-T) for myotubes and 3% BSA in TBS-T for muscle tissue and then incubated overnight at 4°C. Then, the secondary antibodies IRDYE 700 DX-conjugated affinity purified anti-rabbit IgG (H&L) [Donkey] and IRDYE 800-conjugated affinity purified anti-mouse IgG (H&L) [Donkey] (Rockland) were added and incubated for another 30 min. The signal was visualized using Odyssey Infrared Imaging System (Li-Cor Biosciences, Bad Homburg, Germany). Calculation of Western blots densitometry was analysed by using the Image J analysis software (NIH).

### Calculation of the Mean Myofiber Diameter

H&E-labeled 10 µm frozen cross sections derived from five FSHD patients and five age-matched healthy controls were used to calculate myofiber diameters as previously described by Spuler et al. [23]. Ten images (20×magnification) for each FSHD and control subject were captured using a light microscope (Leica Microsystems, Leica DM LB2). Thousand myofibers were chosen to compute mean myofiber diameter. The smallest diameter of myofiber was manually selected and calculated in µm. All computation was done by using the Image J software (version 1.36r; Java 1.6.0_20, Maryland, USA.). To exclude/include the probability of hypertrophy pathway activation, we performed RNA analysis for IGF-1 (regulator of skeletal muscle size) in the FSHD-1 and control groups, but did not find any significant changes. Type 1 and 2 myofibers were distinguished for FSHD-1 patients and healthy controls by ATPase pH 9.4 and ATPase pH 4.6 staining.

### Statistics

RNA expression levels are presented as median, 25th and 75th percentiles and range. Group comparisons were analyzed by using non-parametric Mann-Whitney *U* and Wilcoxon’s rank tests (GraphPad Prism version 4.0 software, San Diego, California, USA).

For the distribution pattern of myofiber diameters (µm) of FSHD patients and healthy controls mean values are given. At least 10 slide fields for each examined subject were selected and examined at a magnification 20x on a Light Microscope (Leica Microsystems, Leica LB2). Group and subject comparisons were analyzed by Wilcoxon test using R: A language and environment for statistical computing (R-Version R-2.15.2, R Foundation for Statistical Computing, Vienna, Austria) [24]. The Histogram was made by use of ggplot2 (Version 0.9.3, Wickham et al. 2009) [25]. Statistical significance was considered at *p*<0.05.

## Results

### Cyclin A1 is Up-regulated at RNA Level in Myotubes and Muscle Tissue from FSHD Patients

**Figure 1:**
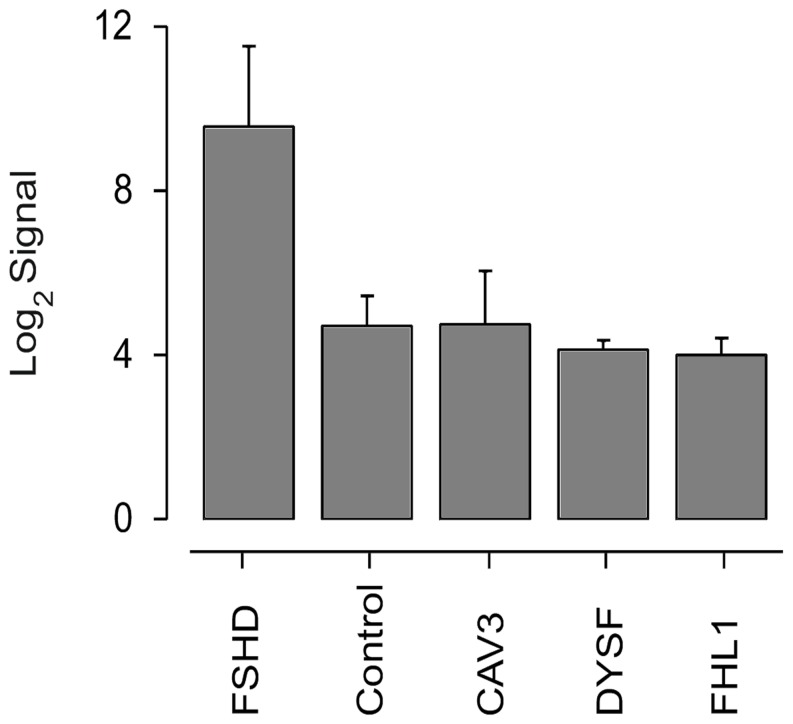
Cyclin A1 expression levels in FSHD and other myopathies (microarrays). *CCNA1* Human Exon 1.0 ST Array signal levels in FSHD (n = 4), healthy controls (n = 7), CAV3 (n = 4), DYSF (n = 4) and FHL1 (n = 3).

**Figure 2:**
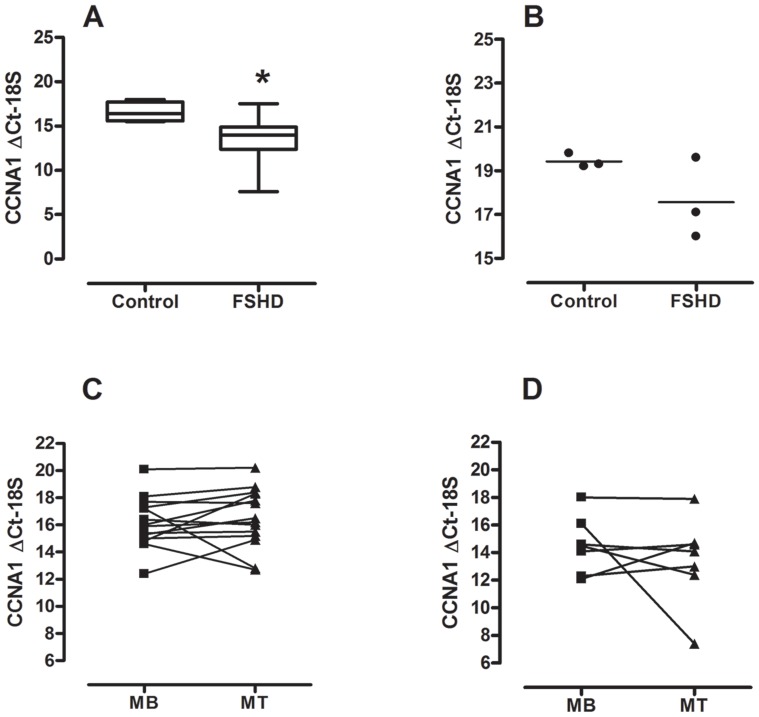
Cyclin A1 expression levels in FSHD (RT-PCR). (**A**) *CCNA1* ΔCt-18S in myotubes derived from FSHD patients (n = 7) and age-matched healthy controls (n = 7), ΔΔCt = −3.2, n-fold change: 2^−ΔΔCt = ^9.2. Data are given as median, 25^th^ and 75^th^ percentiles and range. *) *p*<0.05; Mann-Whitney *U*-test; (**B**) *CCNA1* ΔCt-18S in muscle tissue derived from FSHD patients (n = 3) and age-matched healthy controls (n = 3), ΔΔCt = −1.9, n-fold change: 2^−ΔΔCt = ^3.7. Data are given as single values; (**C**) *CCNA1* ΔCt-18S in myoblasts (MB) and myotues (MT) from healthy controls (n = 14), ΔΔCt = 0.3. Data are given as median; (**D**) *CCNA1* ΔCt-18S in MB and MT from FSHD patients (n = 7), ΔΔCt = 2.0. Data are given as median.

We performed microarray analysis in order to look for differences in myotube gene expression in FSHD-1, three other muscular disorders, and in healthy controls. Only *CCNA1* (ANOVA FDR = 8.7×10^−3^) and *LEUTX* (Leucine Twenty Homeobox, ANOVA FDR = 2,117×10^−3^) RNA levels were highly up-regulated (>28- and 50-fold, respectively) in FSHD. Importantly, cyclin A1 RNA levels of FSHD were not only higher compared to healthy controls but also to patients with the other muscular disorders: CAV3, DYSF, and FHL1 (**Fig. 1**). RT-PCR confirmed our findings for FSHD and controls. *CCNA1* expression was about 9-fold higher (ΔΔCt = −3.2, n-fold change: 2^−ΔΔCt = ^9.2) in myotubes from FSHD patients (mean age 50, n = 7) vs. age-matched controls (mean age 53, n = 7), *p<*0.05 (**Fig. 2A**). *CCNA1* expression was also 3.7-fold higher (ΔΔCt = −1.9, n-fold change: 2^−ΔΔCt = ^3.7) in muscle tissue from FSHD patients (mean age 55; n = 3) vs. healthy controls (mean age 54; n = 3), n.s. (**Fig. 2B**).

### Cyclin A1 RNA Expression is Similar in Myoblasts and Myotubes

We compared *CCNA1* expression by RT-PCR between myoblasts and myotubes and found similar cyclin A1 RNA expression levels in myoblast and myotubes in both healthy controls (**Fig. 2C**, n = 14) and FSHD patients (**Fig. 2D**, n = 7). However, we noticed a large variability within the changes in *CCNA1* Ct values (myoblasts vs. myotubes) between individual cell lines for both healthy controls and FSHD patients. In healthy controls, there were four instances that showed >2 Ct changes, two up and two down. In FSHD patients, one sample went from Ct = 16 to Ct = 8 while another one went from Ct∼12 to 14. In addition, Ct values for myoblasts showed a large variability, ranging from 12 to 20 in healthy controls and from 12 to 18 in FSHD patients. Similar findings have been reported by Homma et al. [26].

### Cyclin A1 is Overexpressed at the Protein Level in Myotubes and Muscle Tissue from FSHD Patients

**Figure 3:**
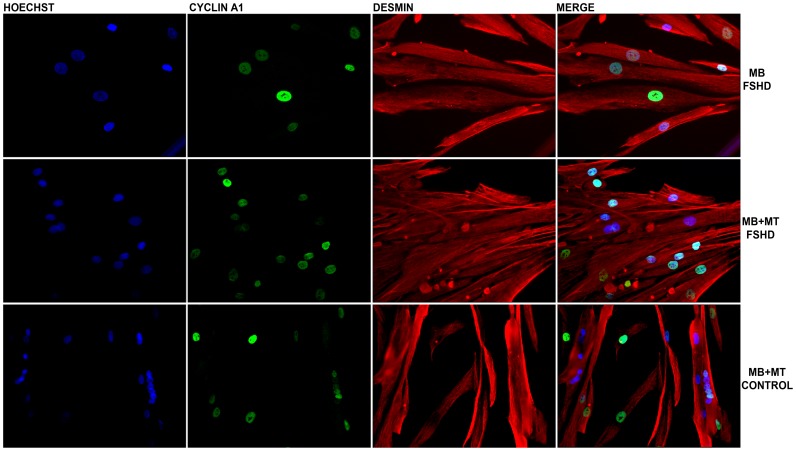
Immunostaining of myoblasts (MB), and myoblasts and myotubes (MB-MT). (40×magnification) from one FSHD patient and one healthy control. Desmin (red), cyclin A1 (green), Hoechst (blue) staining. Cyclin A1 was detected in nuclei of myoblasts and myotubes derived from both FSHD patients and healthy controls.

**Figure 4:**
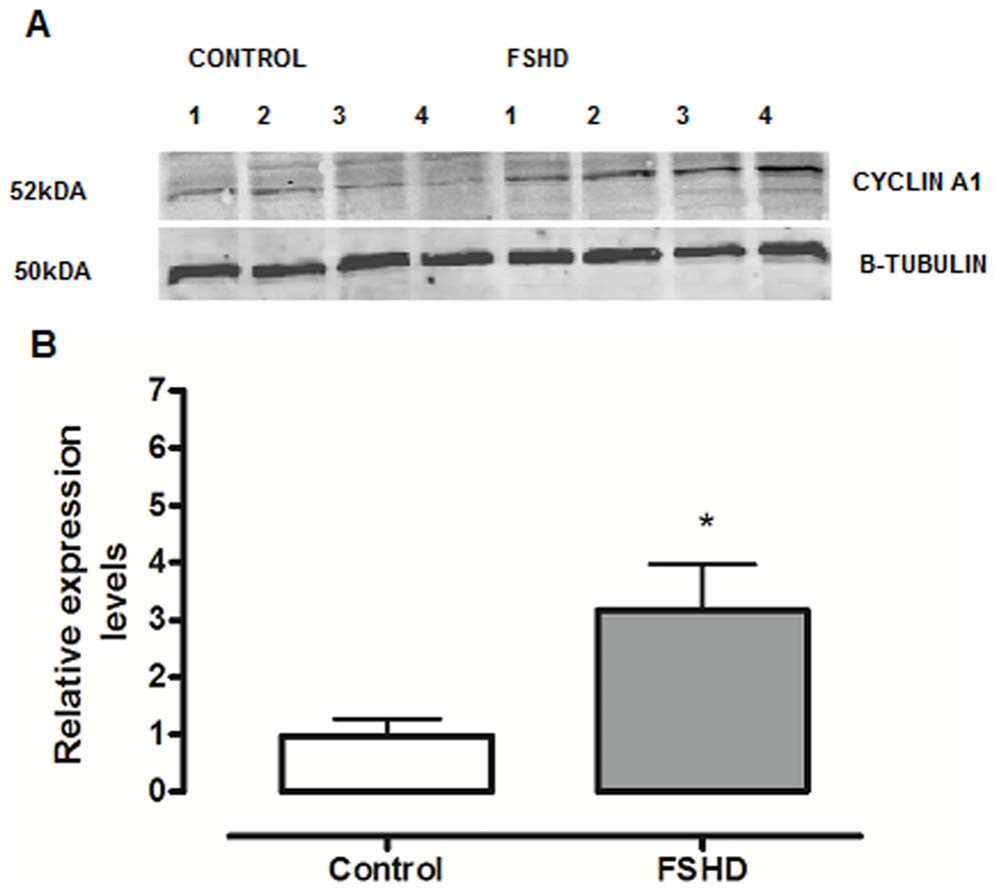
(A) Cyclin A1 protein expression (representative Western blot) in myotubes from FSHD patients (n = 4) and age-matched healthy controls (n = 4). (**B**) Cyclin A1 protein expression (relative changes) in the *Vastus lateralis* muscle from FSHD patients and healthy controls (data derived from A).

**Figure 5:**
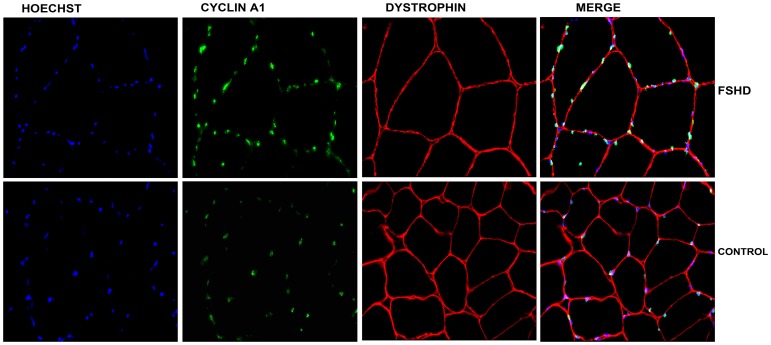
Immunostaining of cryosections of muscle tissue (40×magnification) from one FSHD patient and one age-matched control. Dystrophin (red), cyclin A1 (green), Hoechst (blue) staining.

**Figure 6:**
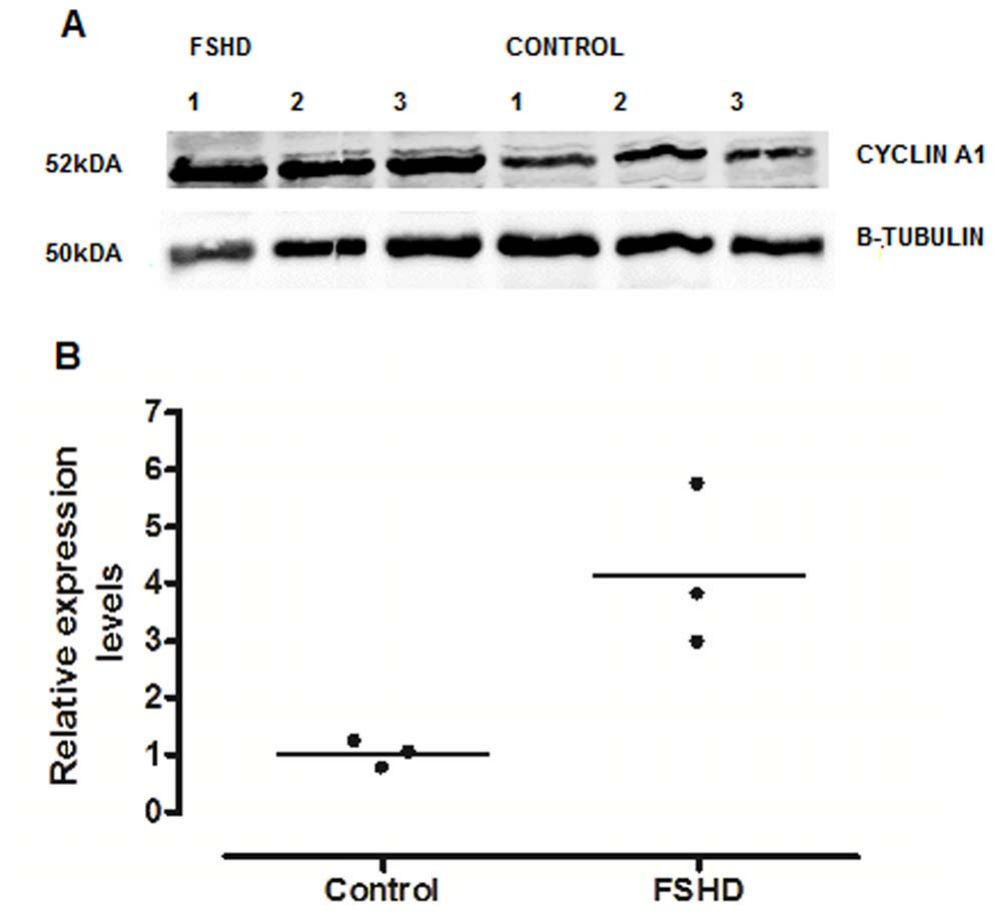
(A) Cyclin A1 protein expression (representative Western blot) in muscle tissue from FSHD patients (n = 3) and age-matched healthy controls (n = 3). (B) Cyclin A1 protein expression (relative changes) in the *Vastus lateralis* muscle from FSHD patients and healthy controls (data derived from A)

We tested for differences in cyclin A1 expression in primary myoblasts and myotubes derived from FSHD-1 patients and healthy controls by using the immunofluorescence technique. Interestingly, we observed in almost all of the stained nuclei a positive cyclin A1 signal (cyclin A1 protein was located in the nucleus, **Fig. 3**). In order to confirm that cyclin A1 is also over-expressed at the protein level in primary myotubes, we performed Western blot analysis and found it clearly up-regulated (>3-fold higher) in FSHD patients (n = 4) vs. age-matched healthy controls (n = 4) (**Fig. 4A, 4B**). These results could be further confirmed by immunohistochemistry in muscle specimens of FSHD patients (mean age 50, n = 7) and age-matched healthy controls (mean age 53, n = 7). Again, most of the myonuclei from both FSHD patients and healthy controls stained positive for cyclin A1 but fluorescence intensity was visually higher in patients vs. healthycontrols (**Fig. 5**). In order to test that cyclin A1 is also over-expressed in muscle tissue, we performed another Western blot analysis and found it also up-regulated (>4-fold) in FSHD patient (n = 3) vs. age-matched healthy controls (n = 3) (**Fig. 6A, 6B**).

### Hypertrophy in FSHD Muscle Tissues

**Figure 7:**
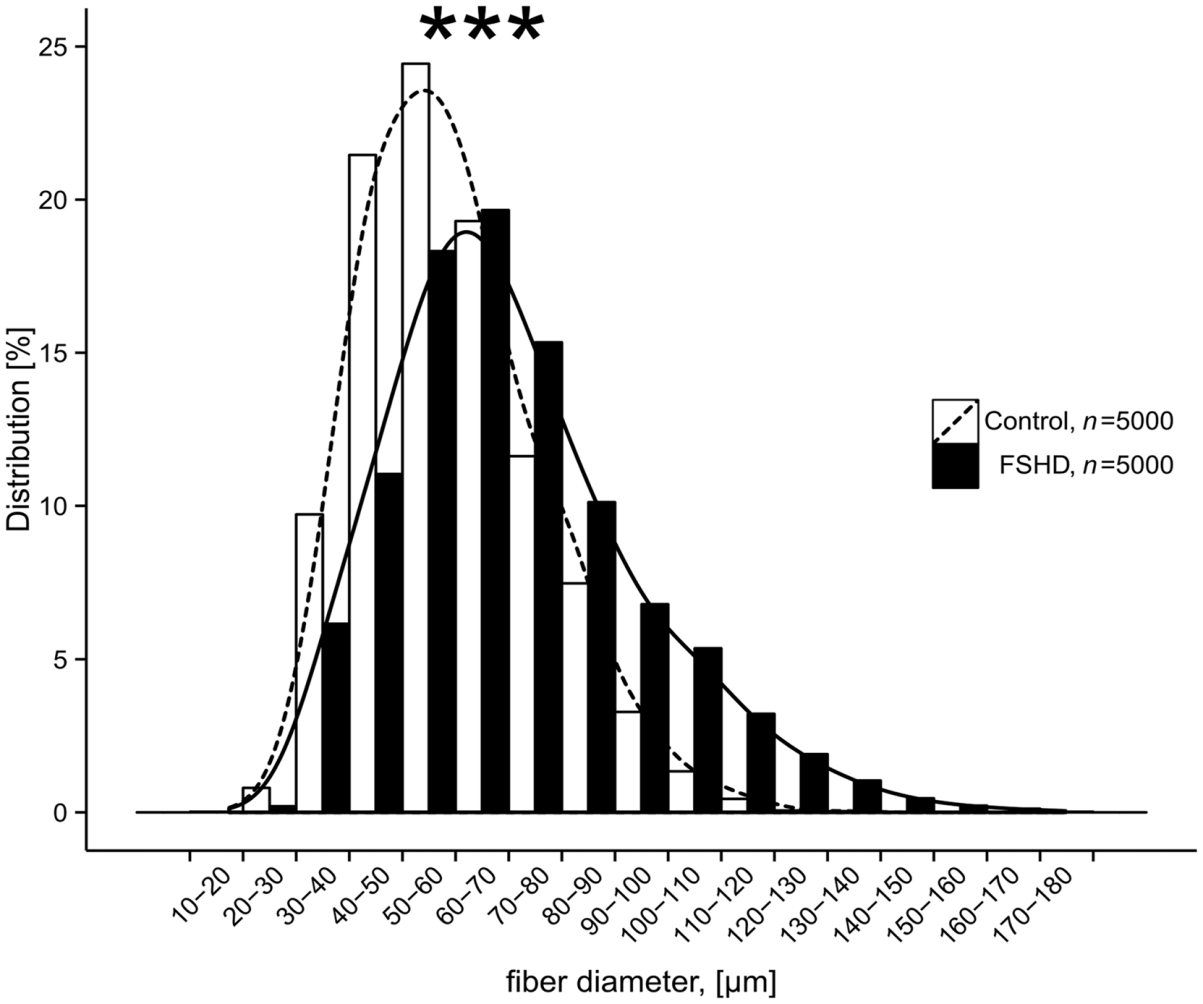
Distribution patterns of myofiber diameters derived from FSHD patients (n = 5) and age-matched healthy controls (n = 5). Diameters (µm) from 1000 myofibers each were measured in H&E-stained cross sections under a light microscope by using the Image J software. Cumulative data are presented (FSHD: mean 71 µm, SD 23 µm; Control: mean 59 µm, SD 17 µm), *p*<0.001, Wilcoxon test.

Muscle fiber diameter was determined on 1000 fibers each (*Vastus lateralis* muscle) from FSHD patients (n = 5) and age-matched healthy controls (n = 5). In controls, the majority of muscle fibers had diameters between 49–59 µm which is consistent with the average fiber diameter of *Vastus lateralis* in healthy subjects calculated by Spuler et al. [23]. In FSHD patients, we found a significant number of fibers with a diameter >120 µm. Mean myofiber diameters were significantly different between the two groups (*p<*0.001, **Fig. 7** **,** **8** **)**. No differences in fiber diameter were found between type 1 and 2 muscle fibers.

**Figure 8:**
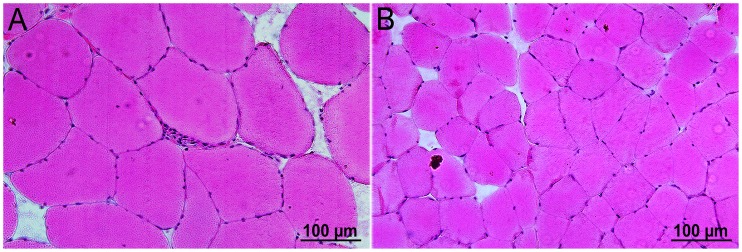
Cross sections of muscle fibers of (A) one FSHD patient and (B) one healthy control (20×magnification, H&E-stained). Myofiber diameter size difference indicates hypertrophy in the *Vastus lateralis* muscle of the patient.

## Discussion

Cyclin A1 is highly expressed in testis and acute myeloid leukemia where it is involved in male meiosis and cell cycle regulation at the restriction points G1/S and G2/M. Generally, cyclin A1 expression levels are low in most somatic cells [16], [27]. Here we show that cyclin A1 is also expressed in skeletal muscle cells lines and muscle tissue but significantly higher in FSHD patients vs. healthy controls at both RNA and protein level.

Interestingly, both cyclin A1 and DUX4 are classified to be epigenetically repressed in somatic cells, and DUX4-fl (a full-length open reading frame mRNA) is known to be a germline transcription factor specifically expressed in FSHD muscle cell lines and muscle tissue contributing to FSHD pathophysiology [27]. In contrast to that, Jones et al. [28] found DUX4-fl expressed at mRNA and protein level in up to 50% of muscle cells and biopsies derived from non-FSHD individuals meaning that it is not sufficient to induce muscle pathology in FSHD.

In FSHD, many genes downstream to DUX4 are activated. There are two transcripts of DUX4, the already mentioned DUX4-fl and DUX4-s, an internally spliced form of DUX4 mRNA. *CCNA1* is downstream to both DUX4-fl and DUX4-s and its expression is obviously 3-fold or even more increased in FSHD patients vs. healthy controls [27]. Recently, it has been reported that cyclin A1 is also up-regulated at RNA level in human immortalized contracted FSHD vs. non-contracted cells [29]. Therefore, our data confirm these findings.

Due to the fact that cyclin A1 is a DUX4-induced protein its inappropriate activation may enter cell cycle processes in the post-mitotic muscle tissue. Interestingly, other highly differentiated post-mitotic cells, for example adult central nervous system neurons, keep their cell cycle in check and re-initiate it at the risk of neurodegeneration [30]. We think a similar process might occur in dystrophic muscle which could indicate a putative role of cyclin A1 in the regeneration processes. Elevated cyclin A1 levels lead to chromatin condensation and apoptosis in renal, ovarian and lung carcinoma cells [31]. Therefore, cyclin A1 could be involved in a way that it controls chromatin de-condensation present in both FSHD-1 and FSHD-2. However, a role of cyclin A1 as a hypothetical protein involved in chromatin formation leaves a question mark and needs to be determined in the future. Furthermore, apoptosis in FSHD occurs at a very low level and cyclin A1 does not seem to play a crucial role here [32].

FSHD primary cell lines are heterogeneous and often contaminated with fibroblasts. In human embryonic fibroblasts, cyclin A1 is highly expressed but redundant [33]. Therefore, we studied *CCNA1* expression in muscle tissue cultures of FSHD and control subjects and found comparable results at the RNA level. In our microarray assays, *CCNA1* expression was detectable at very low levels in healthy controls as well as in some other myopathies such as CAV3, DYSF and FHL1. We could confirm these data in all FSHD patients and healthy controls by RT-PCR analyses. Even if *CCNA1* was detectable at very low levels it was still significantly up-regulated in FSHD patients vs. healthy controls. Interestingly, during myotubes formation, percentage of fibroblast decreased (our own observation) leading finally to an almost pure, high-quality culture of differentiated cells (myotubes). Furthermore, results from RT-PCR, where total RNA was isolated from fresh tissue biopsy, confirmed tissue culture experiment. Nevertheless, cyclin A1 is known to be expressed in hematopoietic progenitor cells and we cannot exclude that its increased expression in our study derives from these cells within our biopsy samples [33], [34].

Progression of FSHD along different muscles shows considerable differences and is obviously patient-specific. In our study, the site of muscle biopsy was chosen for clinical reasons and usually mildly weak muscles (grade 4– affected muscles of the 0–5 Medical Research Council Scale [35]) were biopsied. Still, the material used in this study was heterogeneous. Differences between FSHD cell lines refer to different time points of myoblast growth, expansion and myotube formation in tissue culture, well-known among those working on FSHD. RNA expression levels of cyclin A1 were almost similar in FSHD myoblasts and myotubes within the same patient. Nevertheless, we observed large individual differences of CCNA1 mRNA expression between myoblasts and myotubes and also a large variability of Ct values in myoblasts in both FSHD and control cell lines. This could be due to culturing conditions commonly known in FSHD or, alternatively, an individual variability among subjects as reported by Homma et al. [26].

Results corresponding to the increased muscle myofiber diameter in FSHD are ambiguous. In animal models, muscle hypertrophy was reported and referred mainly to the increase in muscle protein mass. In patients, pseudo-hypertrophy was reported and attributed mainly to deposition of fat and connective tissue [36], [37]. We did not observe significant changes in IGF-1 RNA levels (data not shown) in the whole group but we found significantly (*p<*0.001) increased muscle fiber diameters in FSHD-1 patients vs. healthy controls. Magnetic resonance images could help us to exclude/include the presence of fat and connective tissue infiltration resulting in pseudo-hypertrophy. From our data, we can only speculate that myofiber diameter increases in non-affected muscles to compensate for atrophy of affected muscles in FSHD patients.

Taken together, increased cyclin A1 protein expression could correspond to the increase in muscle protein mass, indicating, for example, disturbances in protein turnover finally leading to muscle hypertrophy [38], [39]. The relation between cell cycle disruption and hypertrophy has not yet been shown in FSHD. Interestingly, one of the ways to counteract excessive cell cycle activation is entering the process of apoptosis [32], [38]–[40]. Therefore, the relation between cell cycle activation, hypertrophy and apoptosis as processes controlling protein mass should be addressed in future studies.

## Conclusion

Cyclin A1 is overexpressed in FSHD at both RNA and protein level. However, the functional role of cyclin A1 overexpression in FSHD remains unknown. The relationship between up-regulated cyclin A1 expression and disease severity and activity needs also to be elucidated. Therefore, we will focus our attention on cyclin A1 and its possible role as a molecular biomarker and indicator of FSHD progression.
